# Supplementary material for: Low Prognostic Nutritional Index Predicts In-Hospital Complications and Case Fatality in Patients with Spontaneous Intracerebral Hemorrhage: A Retrospective Study
Source: Nutrients. 2024 Jun 12;16(12):1841. doi: 10.3390/nu16121841 (PMC11206377; doi:10.3390/nu16121841)
Supplement: Supplementary file 1 [file nutrients-16-01841-s001.zip › nutrients-2984362-supplementary.pdf]

Supplementary Table S1

The demographics of patients who were excluded due to incomplete vital sign and biochemical data.

**Variables**	**Valid n in Exclusion**	**Exclusion**	**Inclusion**	***p*-Value**
Characteristics number		2140	2402	
Ages (years)	2140	59.6 ± 19.7	63.6 ± 18.6	<0.001
Gender, male , n (%)	2140	1160 (54.2%)	1419 (59.1%)	<0.001
BMI	1758	23.3 ± 4.6	23.9 ± 7.5	<0.001
GCS at admission [median (IQR)]	773	13 (8, 14)	13 (9, 14)	0.001
Vital sign at admission				
SBP (mmHg)	786	130.5 ± 17.1	131 ± 16.7	0.446
DBP (mmHg)	786	72.8 ± 10.9	72.9 ± 10.8	0.844
Heart rate(bpm)	786	80.5 ± 14.4	80.1 ± 14.3	0.446
Body temperature (° C)	786	36.6 ± 0.6	36.6 ± 0.5	0.951
Respiratory rate (bpm)	786	17.6 ± 2.3	17.8 ± 2.3	0.018
SPO2 (%)	786	97.8 ± 1.9	97.7 ± 2.1	0.060
Laboratory data at admission				
Creatinine (mg/dl)	1001	1.1 ± 1.6	1.1 ± 1.3	0.450
Hb (g/dl)	1067	12.9 ± 2	13 ± 2.1	0.489
K (mmol/l)	1111	3.8 ± 0.5	3.8 ± 0.5	0.199
Na (mmol/l)	972	137.7 ± 3.6	137.2 ± 3.8	0.000
BUN (mg/dl)	516	18.7 ± 16.6	18.3 ± 12.1	0.619
RBC (10 × 6/μl)	959	4.4 ± 0.7	4.3 ± 0.8	0.020
RDW (%)	959	14 ± 1.6	14.1 ± 1.7	0.135
Hba1c (%)	474	6.6 ± 1.5	6.3 ± 1.1	<0.001
Albumin (g/dl)	242	3.4 ± 0.6	3.4 ± 0.5	0.713
Platelet (10 × 3/μl)	971	214.8 ± 75.3	208.5 ± 79.8	0.034
WBC (10 × 3/μl)	972	9 ± 3.9	10.1 ± 4.8	<0.001
Lymphocyte (%)	931	21.1 ± 11.8	19.6 ± 11.9	0.001
Charlson Comorbidity Index	2140	1.1 ± 2.1	1.9 ± 2.6	<0.001
Medical history				
Antihypertension treatment, n (%)	2140	418 (19.5%)	795 (33.1%)	<0.001
Lipid-lowering treatment, n (%)	2140	165 (7.7%)	331 (13.8%)	<0.001
Non-insulin antihyperglycemic treatment, n (%)	2140	135 (6.3%)	281 (11.7%)	<0.001
Insulin treatment, n (%)	2140	53 (2.5%)	125 (5.2%)	<0.001
NG insertion during admission, n (%)	2140	221 (10.3%)	409 (17%)	<0.001

Supplementary Table S2

The actual data and statistics of all entered variables from our study.

	**Complication**		**28-Day Mortality**		**90-Day Mortality**	
	**aHR (95% CI)**	***p*-Value**	**aHR (95% CI)**	***p*-Value**	**aHR (95% CI)**	***p*-Value**
PNI42_77	0.78 (0.61, 0.99)	0.045	0.73 (0.56, 0.96)	0.023	0.72 (0.56, 0.93)	0.012
Ages	1.01 (1, 1.02)	0.115	1.02 (1.01, 1.03)	<0.001	1.02 (1.01, 1.03)	<0.001
SEX, Male	1.26 (0.94, 1.68)	0.126	1.2 (0.9, 1.59)	0.212	1.2 (0.91, 1.57)	0.194
RBC	0.81 (0.62, 1.06)	0.117	0.9 (0.71, 1.15)	0.418	0.88 (0.69, 1.11)	0.285
Hb	0.96 (0.87, 1.06)	0.477	1.02 (0.94, 1.12)	0.593	1.02 (0.94, 1.11)	0.685
WBC	1.02 (0.99, 1.05)	0.112	1.02 (1, 1.04)	0.102	1.02 (0.99, 1.04)	0.206
Platelet count	1.01 (0.99, 1.03)	0.265	0.98 (0.97, 1)	0.083	0.99 (0.97, 1)	0.125
Creatinine	0.99 (0.86, 1.15)	0.923	1.09 (0.99, 1.19)	0.068	1.11 (1.02, 1.21)	0.014
BUN	0.99 (0.97, 1.01)	0.243	1 (0.99, 1.02)	0.553	1 (0.99, 1.01)	0.702
K	1.02 (0.78, 1.32)	0.904	0.8 (0.64, 0.99)	0.041	0.82 (0.66, 1.01)	0.061
Na	1.02 (0.98, 1.05)	0.275	1.02 (0.99, 1.04)	0.319	1.02 (0.99, 1.05)	0.231
HbA1c	0.93 (0.82, 1.07)	0.316	0.97 (0.86, 1.09)	0.606	0.97 (0.87, 1.09)	0.626
SBP	0.99 (0.98, 1.01)	0.316	0.98 (0.97, 0.99)	0.001	0.98 (0.97, 0.99)	0.000
DBP	0.99 (0.97, 1.01)	0.232	0.98 (0.96, 1)	0.038	0.98 (0.97, 1)	0.046
Heart rate	1.01 (1, 1.03)	0.011	1.04 (1.03, 1.05)	<0.001	1.04 (1.03, 1.05)	<0.001
Body temperature	1.41 (1.11, 1.8)	0.005	0.71 (0.59, 0.84)	0.000	0.69 (0.58, 0.82)	<0.001
Respiratory rate	0.93 (0.88, 0.99)	0.016	1.03 (0.99, 1.08)	0.169	1.03 (0.99, 1.07)	0.203
SpO2	1.03 (0.97, 1.08)	0.352	0.94 (0.91, 0.96)	<0.001	0.94 (0.91, 0.96)	<0.001
CCI_score	1.05 (0.99, 1.12)	0.121	1.03 (0.97, 1.09)	0.316	1.05 (0.99, 1.1)	0.084
Hypertension	0.7 (0.48, 1.02)	0.062	0.9 (0.64, 1.27)	0.552	0.89 (0.64, 1.25)	0.508
Hyperlipidemia	1.26 (0.75, 2.11)	0.379	1.09 (0.67, 1.77)	0.741	1.14 (0.72, 1.81)	0.583
Diabetes mellitus	1.16 (0.66, 2.03)	0.607	1.14 (0.68, 1.92)	0.625	0.98 (0.59, 1.61)	0.923
NG insertion	1.65 (1.16, 2.34)	0.005	1.79 (1.35, 2.37)	<0.001	1.72 (1.31, 2.26)	<0.001
**Glasgow Coma Scale**						
**GCS < 5**	1		1		1	
**GCS: 5–9**	0.28 (0.18, 0.45)	<0.001	0.29 (0.17, 0.48)	<0.001	0.29 (0.17, 0.48)	<0.001
**GCS: 10–12**	0.1 (0.07, 0.15)	<0.001	0.02 (0.01, 0.06)	<0.001	0.05 (0.02, 0.09)	<0.001
**GCS ≥ 13**	0.02 (0.01, 0.03)	<0.001	0.01 (0, 0.02)	<0.001	0.02 (0.01, 0.03)	<0.001
